# Thyroid Disease in Horses—Retrospective Case Series on Patients Examined for Thyroid Disease in an Equine University Clinic (2009–2024)

**DOI:** 10.3390/vetsci12121127

**Published:** 2025-11-27

**Authors:** Sabita Diana Stoeckle, Hannah Julia Stage, Heidrun Gehlen

**Affiliations:** Equine Clinic: Surgery and Radiology, Freie Universität Berlin, 14163 Berlin, Germany

**Keywords:** thyroid gland, goitre, hemithyroidectomy, thyroid adenoma, hypothyroidism

## Abstract

Thyroid diseases in horses are rare and pose challenges for veterinarians due to the complexity of clinical and diagnostic approaches. This retrospective case series describes cases of equine thyroid disease in a referral hospital population. Files of horses examined for suspected thyroid disease at an equine university clinic between 2009 and 2024 were reviewed. Data from nine horses examined for suspected thyroid disease, including signalment, clinical presentation, laboratory results, imaging findings, fine needle aspirates, biopsies, diagnoses, and treatments, were summarised. Diagnoses included thyroid adenoma (*n* = 6), multiple thyroid cysts (possibly thyroid adenoma (*n* = 1)), non-thyroidal illness syndrome (NTIS, *n* = 1), and iodine deficiency goitre (*n* = 1). Diagnostic modalities varied, with, besides manual palpation performed in every patient, basal thyroid hormone measurement and ultrasonography being the most common. Low serum iodine concentrations were noted in four horses, yet iodine supplementation was inconsistently applied. Hemithyroidectomy was performed in four horses, enabling histopathological diagnosis (three out of four). Despite being rare, thyroid diseases in horses require consistent and thorough diagnostic approaches, including imaging, laboratory, and histopathological examinations. Improved screening tools and research could enhance the diagnostic accuracy and management of equine thyroid disorders.

## Simple Summary

Thyroid problems are uncommon in horses and diagnosing them can be difficult because many signs are vague and the tests are not always straightforward. In this study, veterinarians looked back at nine horses that were examined for suspected thyroid disease at a university clinic between 2009 and 2024. They reviewed each horse’s basic information, symptoms, blood tests, ultrasound findings, tissue samples, final diagnoses, and treatments. Most horses had a benign thyroid tumour (adenoma), while others had thyroid cysts, low iodine levels, or illness-related hormonal changes. Ultrasound and thyroid hormone tests were the most frequently used diagnostic tools, and some horses had surgery to confirm the diagnosis. Although thyroid disease is rare in horses, these cases show that careful testing—using imaging, lab work, and tissue examination—is important. Better diagnostic methods and further research could help veterinarians identify and treat thyroid problems more effectively.

## 1. Introduction

### 1.1. Anatomy and Physiology of the Thyroid Gland

The thyroid gland plays a central role in energy metabolism, growth and thermal regulation of the body. The thyroid hormones thyroxine (T4) and triiodothyronine (T3) are crucial for the regulation of many physiological processes [1]. These hormones are reversibly bound to transport proteins, and only the free, unbound fraction of the hormones can cross the capillary endothelium and exert their biological function [1,2].

The thyroid gland, which is not physiologically visible in horses, consists of two lobes which are connected through a fibrous isthmus. In rare cases, the gland may be visible without enlargement. It is located dorsolaterally of the first to third (fourth) tracheal rings and at an angle between the confluence of the linguofacial and maxillary vein [3]. Though not visible, it can be palpated in most horses. The lobes have a firm structure and are movable [3]. The thyroid gland in an adult horse measures approximately 2.5 × 2.5 × 5 cm [1].

### 1.2. Main Thyroid Diseases in Horses

Non-thyroidal illness syndrome (NTIS), also known as Euthyroid Sick Syndrome, describes a change in thyroid hormone concentrations as a result of serious illness without a primary thyroid disorder. In horses with NTIS, a reduction in T3 and T4 with the absence of elevated TSH is commonly observed [4]. This is considered part of the physiological response indicating hypothalamic–pituitary axis dysregulation to systemic diseases such as colic, infections, or sepsis [4]. However, NTIS can also occur in chronic diseases such as pituitary pars intermedia dysfunction (PPID). The aim of this hormonal adaptation is to reduce energy consumption by slowing down the metabolism. NTIS is a secondary change and does not require direct treatment of the thyroid gland but rather the primary treatment of the underlying disease [4].

Hypothyroidism in horses has been linked to different conditions such as altered lipid metabolism [5], agalactia [6], keratoconjunctivitis sicca [7], and alopecia [8]. In a previous study, six horses (four Thoroughbred racehorses, two Standardbred racehorses) were diagnosed with hypothyroidism by low T4 concentrations. These horses presented with poor performance, erratic appetite, decreased endurance, dullness, and stiffness of gait. Additionally, two of the horses had a history of clinically evident exertional rhabdomyolysis [9]. In four out of five donkeys, hypothermia was linked to histological changes compatible with hypothyroidism [10].

A study comparing six thyroidectomised horses with five controls reported that thyroidectomised animals showed less growth, sensitivity to cold, late shedding of the winter coat and coarse hair coat, docile and lethargic behaviour, a sleepy facial expression, oedema of the hind limbs, and a coarse and thickened appearance of the face. Additionally, this study reported decreased feed consumption and body weight gain, especially in mares [11].

A case report on equine hyperthyroidism describes clinical signs such as emaciation, hyperexcitability, polyphagia, tachycardia, polydipsia, enophthalmos, and unilateral thyroid gland enlargement [12]. Reported treatments include hemithyroidectomy and the administration of propylthiouracil [12,13].

### 1.3. Diagnostic and Therapeutic Approach

However, the diagnosis of thyroid dysfunction, which is very rare in horses, is difficult and requires careful interpretation of the clinical signs and laboratory values [1]. Changes in the size of the thyroid gland are common in horses, as are altered concentrations of T3 and T4. In addition to thyroid disease, the basal concentrations of thyroid hormones are also influenced by age, physiological status (pregnancy, lactation), other hormones such as endo- and exogenous steroids, medication, ambient temperature, feed intake, and training [14,15,16,17,18,19,20,21,22,23,24,25,26,27,28,29,30].

Measurement of the Thyroid Stimulating Hormone (TSH) concentration, which is frequently used in human and small animal medicine, is not routinely performed in equine medicine, since there is no commercially available validated assay for equine TSH. Horses with primary (propylthiouracil-induced) hypothyroidism present with decreased concentrations of T3 and T4, as well as increased concentrations of TSH [31].

Stimulation tests (Thyroid-Releasing Hormone (TRH) stimulation test, TSH stimulation test) are used to determine the presence of hypothyroidism [32]. These tests are expensive and time-consuming and are often not used in practice to diagnose thyroid disease.

As recommended by the equine endocrinology group, the TRH stimulation test should be performed if both the T3 and T4 as well as the biologically active free/unbound T4 (fT4) concentrations are decreased. For the TRH stimulation test, 1 mg of TRH is administered intravenously after the collection of serum for the baseline concentrations of T3 and T4. The T3 and T4 concentrations are determined again two and four hours later. Greater than 1.5-fold increases in blood T3 and T4 concentrations are normally detected, although any increase is evidence that the gland is responding to the TRH and should exclude a diagnosis of primary hypothyroidism [33]. In ponies, a dosage of 0.5 mg TRH is commonly used [1].

Even though thyroid dysfunction is rare in horses, thyroid neoplasia seems to be quite common in horses. Thyroid neoplasia is encountered in up to one-third of horses, mostly in horses older than 17 years [34]. The most common thyroid neoplasm is the thyroid adenoma which often only affects one thyroid lobe [12,35,36,37,38]. Most equine thyroid adenomas are non-functional and do not progress to thyroid adenocarcinomas [34]. However, some authors suggested that thyroid adenocarcinoma is the most common thyroid neoplasia [39,40,41]. Due to an increased risk of bleeding, performance of a thyroid biopsy is currently not routinely recommended [33].

### 1.4. Objectives and Relevance of This Work for Equine Medicine

Despite decades of study, many aspects of equine thyroid disease remain unclear. We still do not fully understand how often true primary thyroid dysfunction occurs in horses, since many clinical signs attributed to “thyroid problems” are actually caused by non-thyroidal illness, age, diet, or training status. Additionally, the mechanisms driving rare conditions like autoimmune thyroiditis in horses are poorly defined, and it remains uncertain how—or even whether—thyroid hormone supplementation benefits horses without confirmed glandular disease. Therefore, we analysed our patient data for patients examined for thyroid disease over a time of 15 years.

## 2. Material and Methods

Horses that were presented or referred for examination of the thyroid gland from January 2009 to December 2024 were identified by searching the practice management programme (Vetera, Vetera GmbH, Eltville am Rhein, Germany) with keywords. Additionally, horses with incidental findings at the thyroid gland were included. Since the clinic is located in Germany, mostly German keywords, specifically “Jod (iodine)”, “Schild- (thyroid)”, “Schilddrüse (thyroid gland)”, “T3”, “T4”, “Thyroid”, “-thyreose”, “-thyreot”, and “TSH”, were used. The patients were presented/referred due to suspected thyroid disease by the owner or a local veterinarian.

Abstracted patient specific data included, beside the signalment (breed, age, weight) and date of presentation, the reason for presentation at the clinic; if the horse had a goitre; concentrations of the thyroid hormones T3, T4, and fT4; iodine concentration as measured in the blood; sonographic findings of the thyroid gland; the presence of other diseases, if a fine needle aspirate (FNA) and/or a biopsy was performed and their results; and the diagnosis related to the thyroid gland and the treatment of the thyroid disease.

In cases in which a TRH stimulation test or other imaging studies were performed, these results were abstracted as well.

Due to the retrospective nature of the study, ethical approval was not required. During the admission process at the clinic, horse owners consented to anonymised publication of their horses’ cases.

## 3. Results

### 3.1. Study Population

In total, 9 horses were examined for (suspected) thyroid disease from January 2009 to December 2024.

Two horses presented in 2017, one in 2018, one in 2020, one in 2021, one in 2022, one in 2023, and one in 2024 out of 16305 cases presented to the clinic during that time period. Additionally, one horse presented twice due to unilateral goitre (2021 and 2024). The incidence of horses presented for examination of the thyroid gland in this clinic was therefore 0.06%.

With regard to breed, four horses were warmbloods. The other horses were an American Quarter Horse, a Friesian, a Haflinger crossbreed, a Pura Raza Espagnola (PRE), and a Shetland pony crossbreed. There were five geldings, one stallion, and three mares. The horses were aged 17.8 ± 5.3 years old (mean ± standard deviation, range: 8–25 years). Weight was available for 11/12 horses, and the horses weighed 502.6 ± 153.3 kg (range: 160–695 kg).

**Figure 1:**
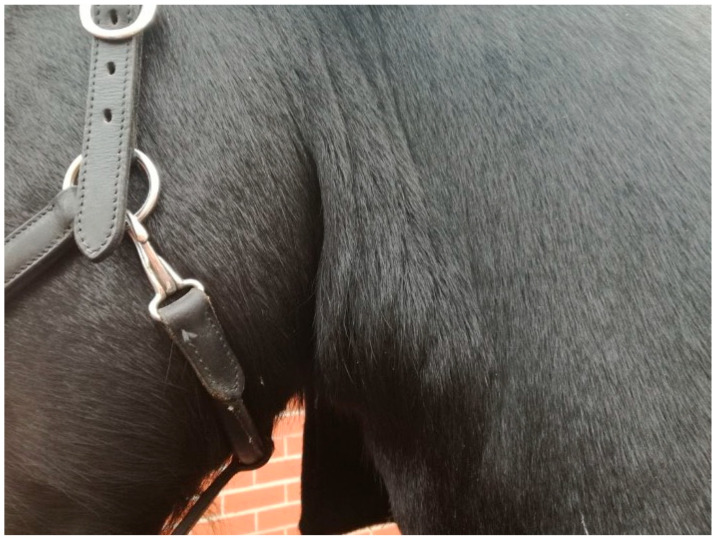
Unilateral goitre in Horse 1.

Reasons for presentation were goitre (unilateral: *n* = 4, bilateral: *n* = 1; Figure 1), poor performance (*n* = 1), chronic laminitis (*n* = 1), colic (*n* = 1), and collapse (the horse was falling down when the head was elevated, *n* = 1).

Diagnosed concomitant diseases during hospitalisation included laminitis (*n* = 2), PPID (*n* = 1), equine asthma (*n* = 2), atrial fibrillation (*n* = 1), mild tricuspid and mitral valve regurgitation (*n* = 1), and insulin dysregulation (*n* = 1).

### 3.2. Clinical Examinations and Laboratory Tests

Upon clinical examination, unilateral goitre was present in 6 horses (*n* = 4 as a presenting complaint, *n* = 2 as an incidental finding) and bilateral goitre in one horse (Table 1).

Thyroid hormone concentrations were measured (T3, T4, fT4) in six horses. In one pony (Horse No. 6), a TRH stimulation test was performed (0.5 mg TRH i.v., determination of T3 and T4 immediately before and 2 h and 4 h after TRH stimulation; Table 2). The results of the stimulation test were supportive of NTIS due to PPID, and the pony also showed laboratory signs of insulin dysregulation (Table 2).

The blood iodine concentration was determined in four horses, and it was decreased in all of these horses (Table 2).

### 3.3. Diagnostic Imaging

**Figure 2:**
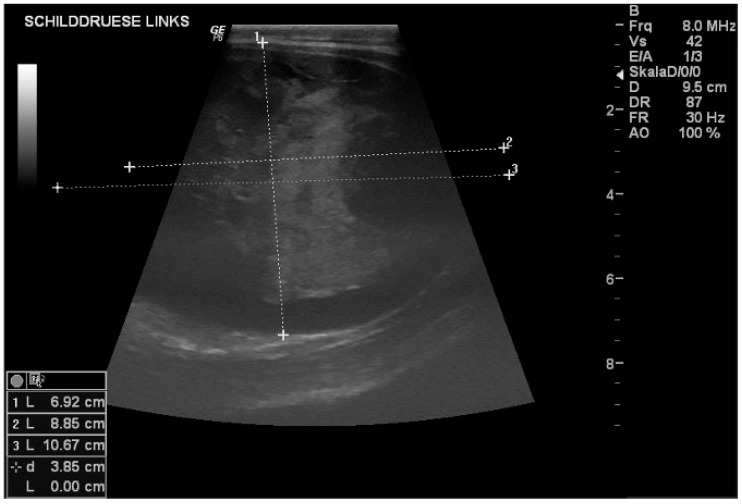
Sonographic imaging reveals a solid enlargement of the left lobe of the thyroid gland. The lesion appears to be well-defined with heterogeneous echotexture. No evidence of cystic components or significant vascularisation. Image obtained with a linear probe with a frequency of 8 Mhz and a depth of 9 cm.

**Figure 3:**
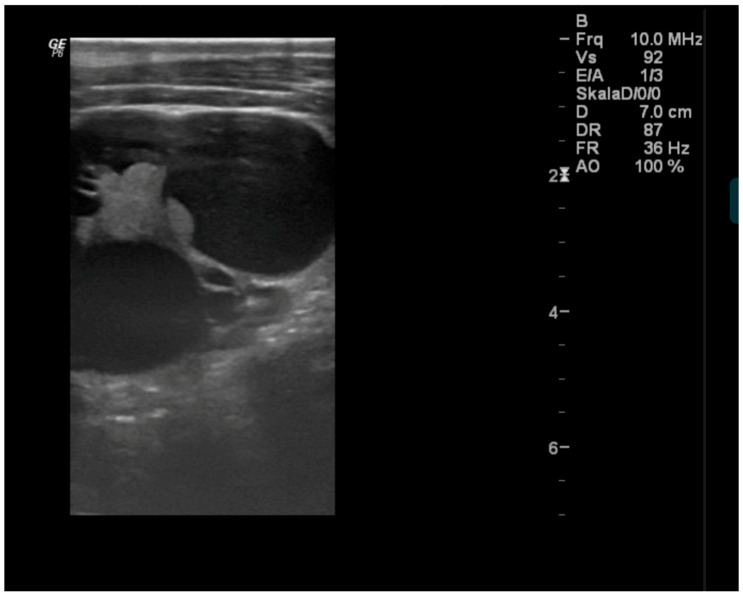
Sonographic image of the right lobe of the thyroid gland in Horse 3 (image obtained in 2021). The lesion appears well-demarcated, with anechoic fluid content. No evidence of solid components or significant vascularisation. Image obtained with a linear probe with a frequency of 10 Mhz and a depth of 7 cm.

Ultrasonography was performed in 8/9 horses. In all horses, both the normal and affected thyroid lobes were scanned, and their size was compared with the reference interval (2.5 × 2.5 × 5 cm). The horses with unilateral goitre mostly had sonographic changes in the enlarged thyroid glands, which were either interpreted as solid (Figure 2) and/or cystoid masses (Figure 3). In the horse with bilateral goitre, no abnormal findings regarding tissue echogenicity were observed (Table 3).

**Figure 4:**
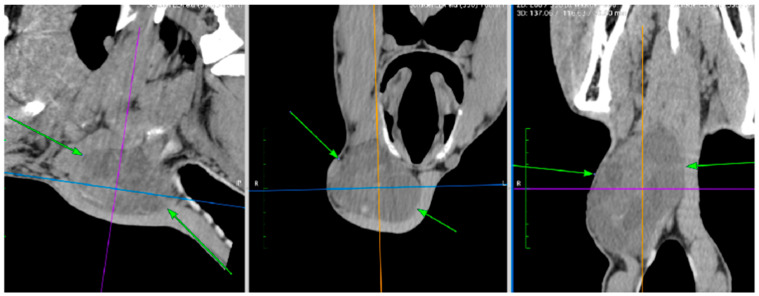
CT-images of Horse 3 (images obtained in 2024), images obtained in the standing horse. A large, well-defined multilobulated cavitary soft tissue mass (12 × 8 × 7 cm, right lobe of the thyroid gland) is present at the right ventral part of the cranial cervical region with several rounded hypodense cavitary pockets (25–35 Hounsfield units ) separated by heterogeneous septa. The mass extends to the midline.

Two horses underwent a CT examination to either determine the extent of the cystoid thyroid changes for improved surgery planning (Horse No. 3, Figure 4) or to identify the reason for collapse since the goitre was not visible externally (Horse No. 8).

### 3.4. Cytopathological and Histopathological Examinations

In four horses, either a fine needle aspiration (*n* = 3) or a biopsy (*n* = 1) was performed. The cytopathological examination led to a (suspected) diagnosis of a thyroid adenoma (*n* = 2, one of the two was a suspected diagnosis, since the cytological image was not entirely typical) or no diagnosis (*n* = 1, horse No. 3). The biopsy identified a thyroid carcinoma, but after the histopathological examination of the removed thyroid lobe, the pathologist revised their diagnosis to thyroid adenoma (Horse No. 1).

In all other four cases in which hemithyroidectomy was performed due to movement and/or breathing restriction and/or rapid growth of the mass, histopathology was performed on the removed thyroid lobe. Three out of four cases were identified as a thyroid adenoma and one out of four cases as multiple thyroid cysts, possibly due to thyroid adenoma (Table 3). The cases with (suspected) thyroid adenoma in which the enlarged thyroid gland did not cause clinical signs such as difficulty breathing and/or swallowing, hemithyroidectomy was not pursued at the owner’s request.

In horses with suspected neoplasm of the thyroid gland in which fine needle aspiration/biopsy and/or hemithyroidectomy was declined, a thyroid adenoma was suspected, since this is the most common neoplasm of the equine thyroid gland (Table 1).

On the basis of the entirety of the examination results, horses were diagnosed with (suspected) thyroid (cyst-)adenoma (*n* = 6), multiple thyroid cysts possibly due to thyroid adenoma (*n* = 1), NTIS (*n* = 1), and iodine deficiency goitre (*n* = 1). Horses were treated with hemithyroidectomy (*n* = 4, one horse with additional iodine supplementation), or iodine supplementation exclusively (*n* = 1). The owners of the remaining horses with iodine deficiency were recommended to adjust the mineral feed to the horse’s requirements.

## 4. Discussion

During the years 2009–2024, only nine horses were examined for thyroid disease, most of which were diagnosed with a (suspected) thyroid adenoma. There was no horse with hypothyroidism. It is remarkable that only during the more current analysed years were horses examined for thyroid disease. Thyroid disease seems to be more common in humans (incidence of hypothyroidism, 0–7%; incidence of hyperthyroidism, 0–8% [42,43,44,45,46]) and small animals (hypothyroidism in dogs, 0.23–0.4% [47,48]; hyperthyroidism in cats, 2–14% [49,50,51]). Due to the recently released “*Recommendations on Diagnosis and Management of Thyroid Disease and the Use of Thyroxine in Horses*” [33] by the equine endocrinology group, referral equine hospitals might be presented with further horses with suspected thyroid disease in the following years.

A retrospective case series on equine thyroid disease adds something genuinely useful to the field because reliable data on these disorders in horses are surprisingly sparse. Most of what clinicians think they know is extrapolated from other species or based on scattered single-case reports, so patterns of presentation, imaging findings, and outcomes remain murky. Pulling multiple cases together lets us see how these conditions actually behave in practice—how they look on ultrasound or CT, how often biopsy changes the diagnosis, and which treatments lead to improvement. Even with its inherent limitations, this case series can map the landscape of a poorly documented disease and gives future studies a clearer place to begin.

The clear limitations of this study are its retrospective nature and therefore the non-standardised examinations of the horses. Additionally, flaws in documentation complicate the retrospective evaluation of the cases. Additionally, analysing the patient data of more than one hospital might have given an improved insight on the incidence of thyroid disease in horses. Furthermore, follow-up of the patients was not pursued.

In this hospital population, the incidence of thyroid disease was particularly low (0.06%), as reported in the literature, where no clear data on the incidence of thyroid disease in horses can be found [1,52]. However, a hospital population hardly presents the entirety of the equine population, and the breed distribution for this cohort was considered typical for horse breeds presenting regularly to the hospital. Since most neoplasms of the equine thyroid gland are non-functional microadenomas [34], many horses with an enlarged thyroid gland are—in the authors’ experience—not referred for evaluation. Therefore, the incidence of thyroid disease in German horses may be much higher than suggested by this case series. NTIS, which occurred in one patient (Horse No. 6) in this case series, may occur secondary to PPID and metabolic syndrome in horses [1]. However, the mechanism of NTIS remains unclear, but thyroid function seems to be adequate in these cases [1]. In horses with endocrinopathies, the altered hormone profile may favour NTIS. The true incidence of NTIS in horses and a possible benefit of thyroid hormone supplementation in these cases remains unknown. In Horse No. 1 with thyroid adenoma and atrial fibrillation, NTIS was not diagnosed, since the thyroid hormone concentrations were normal, apart from a minor decrease in the fT4 concentration. The horse with equine asthma was not examined for NTIS, so it might have been missed in this horse.

Thyroid neoplasia commonly occurs in older horses, with most affected animals being older than 16 years [1]; other authors reported thyroid neoplasia in horses as young as six years old [40,41]. The population we reported on included animals as young as 11 and 12 years, but five out of seven horses with thyroid neoplasia were older than 16 years. In Horse No. 3, a branchial remnant cyst was the main differential after the first hospitalisation; however, after removal of the mass, the histopathological diagnosis was thyroid cysts, possibly a thyroid adenoma. Rarer differentials such as branchial remnant cysts, as described by Slovis et al., should also be considered in cases with a typical sonographic appearance (an anechoic fluid-filled structure with a well-defined hyperechoic capsule) [53]. In four horses, the (suspected) diagnoses was based on FNA (*n* = 3) and ultrasonography (*n* = 1). This clearly limits the diagnostic sensitivity. However, in human medicine, FNA of thyroid tissue has demonstrated good sensitivity. However, ultrasound-guided procedures are more sensitive than those performed using palpation, particularly when thyroid changes—so-called nodules—cannot be palpated or are very small, or when multiple nodules are present [54,55]. However, in humans, thyroid biopsy has a higher adequacy rate than fine-needle aspiration but appears to be less sensitive, especially for papillary carcinoma. According to one study, combining both methods yields the highest adequacy rate and sensitivity [56]. To date, no comparative data are available for horses.

It is remarkable that not all horses that underwent sonography and/or hemithyroidectomy had their thyroid hormone concentrations measured. However, thyroid dysfunction is rare in horses with thyroid neoplasia; therefore, thyroid hormone concentration measurement and dynamic testing might be reserved for cases with suspicious clinical signs as described earlier.

Hemithyroidectomy is usually only recommended in cases with respiratory impairment, gulping, and/or rapid growth of the thyroidal mass [37,39,40,41,57,58]. Since these cases were referred to a university hospital, impairment was likely but was not documented in all cases. Documentation in these cases should be improved to highlight the clinical indication for the surgery.

In complicated cases of thyroid neoplasms, such as extensive tumours or those not externally visible, a standing CT examination may be a useful adjunctive examination, but surely not for the “typical” cases. Cases such as Horse No. 8 are extremely rare and warrant extensive and organised work-up, with the necessity of ruling out other causes for collapse.

However, this will possibly remain an adjunctive imaging modality for thyroid disease in the horse due to increased expense regarding time and cost in otherwise easily and completely worked up cases.

The owners consented to a thyroid biopsy in only one case, but although it provides the veterinarians with valuable results regarding the type of neoplasia, it may be associated with bleeding as the major complication and is therefore not recommended by the equine endocrinology group [33]. Fine needle aspiration may be a less risky alternative but only resulted in a definite diagnosis in one of three cases in this population. Biopsy or fine needle aspiration was recommended in cases in which differentiation of the mass would have altered the decision for or against hemithyroidectomy. In cases in which hemithyroidectomy was indicated due to clinical signs such as movement and/or breathing restriction as well as gulping due to external compression of the larynx and/or trachea [37,39,40,41,57,58] or rapid growth, a biopsy before mass removal was not required, and histopathology was performed after removal of the mass.

The diagnostic work-up of cases also included, in four cases, the measurement of the blood iodine concentration and revealed iodine deficiency in all tested horses. In only two horses, iodine supplementation was included in the discharge instructions; in the other horses, review and, if necessary, adaption of the horses’ mineral feed was recommended. It would have been more appropriate to examine the urinary iodine–creatinine ratio, since this more accurately displays the current iodine supply of the horse, since the serum iodine concentration largely remained the same during different iodine supplementation protocols [59]. Another study claimed that the iodine concentration in equine serum may vary according to location [60]. The serum iodine concentration of mares on the day of foaling (19 ± 0.5 (17–22) µg/L) was reported to be much lower than the reference interval given by the laboratory (50–120 µg/L) [61]. Another study defined horses with a serum iodine concentration of <10 mg/L as iodine-deficient and those with an iodine concentration of 11–19 mg/L as marginally iodine-deficient. They classified horses with a serum iodine concentration of 20–49 mg/L as adequately supplied and horses with a serum iodine concentration >50 mg/L as highly supplied [62]. Comparing these intervals to the serum iodine concentrations of the horses identified as iodine-deficient in this study, maybe only one in four horses was marginally iodine-deficient. The given serum iodine reference intervals might require revision, and it seems questionable if all horses diagnosed as iodine-deficient in this case series were really iodine-deficient.

Unfortunately, TSH assays are not available for commercial testing in horses, as they are in human and small animal medicine which, would makes diagnosis and monitoring easier [1,31].

As expected, this review study shows that thyroid disease in horses is rare, but diagnostic testing is often not pursued in an appropriate manner. Hormone measurements and dynamic testing should be employed as recommended by the equine endocrinology group [33]. Horses with an enlarged thyroid gland should not only be examined clinically and by laboratory diagnostical examinations but also by ultrasonography. Further research and the development of appropriate screening tests could help to improve the diagnostic work-up of these cases.

## Figures and Tables

**Table 1:** Details of the horses and their concomitant diseases.

Horse	Presenting Complaint	Breed	Age (Year)	Body Weight (kg)	Goitre	Concomitant Disease
1	Unilateral goitre	Friesian	12	615	Unilateral	Lameness, atrial fibrillation
2	Poor performance	Warmblood	21	580	Unilateral	Equine asthma
3	Laryngeal swelling	Warmblood	16 (2021) 19 (2024)	550	Unilateral	2024: Tricuspid and mitral valve regurgitation, equine asthma
4	Thyroid examination	Warmblood	8	640	Bilateral	-
5	Mass at the jugular vein	Pura Raza Espagnola	19	540	Unilateral	-
6	Chronic laminitis	Shetland pony crossbreed	21	160	No	PPID, EMS, laminitis
7	Colic	Warmblood	11	695	Unilateral	-
8	Collapse	Haflinger crossbreed	23	475	No	Laminitis
9	Unilateral goitre	American Quarter Horse	16	540	Unilateral	-

**Table 2:** Results of thyroid hormone and blood iodine measurements.

					TRH Stimulation			
Horse	T3 (ng/dL, 25–180 ng/dL)	T4 (µg/dL, 1.3–4.1 µg/dL)	fT4 (pmol/L, 9–44.9 pmol/L)	Iodine (Blood, µg/L; 50–120 µg/L)	T3 After 2 h	T3 After 4 h	T4 After 2 h	T4 After 4 h
1	41.8	1.62	8.9	21	-	-	-	-
2	57.2	1.31	9.2	13.1	-	-	-	-
4	114.2	2.42	14.8	25.4	-	-	-	-
5	145.1	1.73	14.9	25	-	-	-	-
6	33	1.27	-	-	82.9	87.2	1.92	2.28
7	69.6	2.6	13.8	-	-	-	-	-
8	45.5	1.6	8.6	-	-	-	-	-

T3, triiodothyronine; T4, thyroxine; TRH, thyrotrophin-releasing hormone.

**Table 3:** Imaging findings, thyroid diagnosis, and treatment of thyroid disease.

Horse	Sonography	Biopsy/FNA	CT	Diagnosis of Thyroid Examination	Thyroid Treatment
1	Right lobe WNL, left lobe inhomogenous with calcifications, 5.6 × 10 cm	Biopsy: thyroid adenocarcinoma Histopathology after hemithyroidectomy: thyroid adenoma	-	Unilateral thyroid adenoma	Hemithyroidectomy, iodine supplementation
2	Left lobe WNL, right lobe with encapsulated structure, 3.3 × 4.4 cm	-	-	Suspected unilateral thyroid adenoma	None
3	2021: Thyroid WNL, multiple cysts ventral to the thyroid 2024: No normal thyroid tissue visible, cystoid changes	-	2024: Lobulated cystic soft tissue mass in the right ventral cranial cervical region at the level of the larynx	2021: Suspected branchial remnant cyst 2024: Multiple thyroid cysts, thyroid adenoma cannot be excluded	2021: None 2024: Hemithyroidectomy
4	Left lobe 4.14 × 2.74 cm, right lobe 4.47 × 1.87 cm, homogenous tissue	-	-	Iodine deficiency struma	Iodine supplementation
5	Right lobe 3.5 × 3.8, left lobe 2.1 × 3.5 cm, homogenous tissue	FNA: Suspected benign thyroid neoplasia	-	Unilateral euthyroid adenoma	None
6	-	-	-	NTIS	None
7	Right lobe with round to ovoid structures, no capsula, 4–6 cm, outer tissue heterogenous, two structures anechogenic fluid in the centre, chambered, single hyperechogenic spots	FNA: Suspected benign thyroid neoplasia	-	Suspected unilateral thyroid adenoma	None
8	Right lobe: 12 × 10 cm heteroechogenic mass	-	Right lobe: with lobulated structure 5.8 × 3.5 × 2.3 cm, inner structure soft tissue dense and ovoid, lateral area with 1 cm large, spherical, hypodense area; left lobe: homogenous soft tissue dense, ovoid 3.9 × 2.2 × 1.8 cm	Unilateral euthyroid cystadenoma	Hemithyroidectomy
9	Left lobe WNL, right lobe 7 × 7cm, tissue inhomogenous, encapsulated, vascularised, few anechogenic chambers	-	-	Suspected unilateral thyroid adenoma	Hemithyroidectomy

WNL, within normal limits; FNA, fine needle aspirate; NTIS, non-thyroidal illness syndrome.

## Data Availability

The original contributions presented in this study are included in the article material. Further inquiries can be directed to the corresponding author.
